# RETRACTED: Lee et al. Myogenesis Effects of RGX365 to Improve Skeletal Muscle Atrophy. *Nutrients* 2023, *15*, 4307

**DOI:** 10.3390/nu16193412

**Published:** 2024-10-09

**Authors:** Hye-Jin Lee, Hui-Ji Choi, Sang-Ah Lee, Dong Hyuk Baek, Jong Beom Heo, Gyu Yong Song, Wonhwa Lee

**Affiliations:** 1 Department of Chemistry, Sungkyunkwan University, Suwon 16419, Republic of Korea; 2 College of Pharmacy, Chungnam National University, Daejeon 34134, Republic of Korea; 3 Faculty of Biotechnology, College of Applied Life Sciences, Jeju National University, Jeju 63243, Republic of Korea; 4 Environmental Safety Group, Korea Institute of Science and Technology (KIST) Europe, 66123 Saarbruecken, Germany; 5 AREZ Co. Ltd., Daejeon 34036, Republic of Korea

The journal retracts the article, “Myogenesis Effects of RGX365 to Improve Skeletal Muscle Atrophy” [1], cited above.

Following publication, concerns were brought to the attention of the publisher regarding a number of image duplications contained within this publication [1].

Adhering to our standard procedure, an investigation was conducted by the Editorial Office and Editorial Board that confirmed the presence of duplicated panels contained within Figures 1C and 4A, presenting different experimental conditions. While the authors cooperated with the investigation, they were unable to satisfactorily explain these duplications nor were they able to provide verifiable supplementary material for evaluation by the Editorial Board. As a consequence, the Editorial Board have lost confidence in the validity of the findings and decided to retract the publication [1] as per MDPI’s retraction policy (https://www.mdpi.com/ethics#_bookmark30 (accessed on 20 September 2024)).

This retraction was approved by the Editor-in-Chief of the journal *Nutrients*.

The authors did not agree to this retraction.
